# Changes in Access to Alcohol-Based Hand Rub and Hand Hygiene Adherence among Healthcare Workers after a Hand Rub Production and Distribution Program in Rural Uganda before and during the COVID-19 Pandemic

**DOI:** 10.4269/ajtmh.24-0040

**Published:** 2024-09-18

**Authors:** Kanako Ishida, Matthew Lozier, Alexandra M. Medley, Victoria Trinies, Christiana Hug, Carrie Ripkey, Maureen Kesande, Fred Tusabe, Sauda Yapswale, Francis Ocitti, Herbert Isabirye, Judith Nanyondo, Martin Watsisi, Mohammed Lamorde, David Berendes

**Affiliations:** ^1^ Division of Foodborne, Waterborne, and Environmental Diseases, National Center for Emerging and Zoonotic Infectious Diseases, Centers for Disease Control and Prevention (CDC), Atlanta, GA;; ^2^ CDC Foundation, Atlanta, GA;; ^3^ Infectious Disease Institute, Makerere University, Kampala, Uganda;; ^4^ IRC International Water and Sanitation Centre, Kabarole District, Uganda

## Abstract

During the COVID-19 pandemic, the use of alcohol-based hand rubs (ABHRs) was critical for improving hand hygiene (HH) among healthcare workers (HCWs). Before and during the pandemic, we supported district-led production and district-wide distribution of ABHRs and one-time provision of portable handwashing stations to select healthcare facilities (HCFs) in five rural districts in Uganda. Comparison between baseline and follow-up assessments showed an overall increase in access to HH materials and HH adherence (HHA; handwashing with soap and water or use of ABHR) among HCWs. However, large differences in the changes in HH material coverage and HHA across districts may have been heavily influenced by the COVID-19 disease burden and its risk perception when the assessments were conducted. Using data collected at multiple time points before and during the pandemic across districts and estimating and controlling for pandemic effects in an exploratory multivariate analysis, the adjusted odds ratio of HHA in district HCFs was 4.6 (95% CI: 1.8–11.8) after (versus before) the ABHR intervention. This increase appeared to be primarily in larger HCFs, where the perceived need for ABHRs may have been greater. Additional strategies are needed to further increase HHA, especially in the smallest HCFs, among laboratory technicians and nurses and before patient contact. However, district-scale ABHR interventions seemed successful in ensuring the continued availability of HH materials.

## INTRODUCTION

Hand hygiene adherence (HHA), defined as handwashing with soap and water or using alcohol-based hand rub (ABHR), is a key prevention measure recommended to reduce the infectious disease burden worldwide, especially during the COVID-19 pandemic.^1^^,^^2^ Hand hygiene adherence among healthcare workers (HCWs) is particularly important to reduce disease transmission in healthcare settings.^3^^–^^5^ As a low-income country,^6^ Uganda has multiple obstacles to improving water, sanitation, and hygiene (WASH) coverage in its healthcare facilities (HCFs). In particular, the availability of hand hygiene (HH) resources at points of care or at toilets at HCFs in Uganda remains low, with estimates ranging from 20%^7^ to 24%.^8^ An increased use of ABHR at points of care can effectively and rapidly improve HH and help control healthcare-associated infections,^9^^,^^10^ including COVID-19.

Many HCFs in low- and middle-income countries are financially unable to purchase commercial ABHRs; thus, local production may be a more economical option.^11^ The WHO has published guidance and formulations for producing ABHRs,^12^^,^^13^ and local production of ABHRs has been implemented within individual HCFs, often hospitals, in several countries and by private companies or government agencies for distribution to HCFs.^14^ Alcohol-based hand rub production is rarely feasible for smaller HCFs that may be located in areas where access to commercial ABHRs is also limited. We instituted centralized ABHR production at large HCFs in five districts with predominantly rural populations in Uganda coupled with district-wide distribution to select government and private not-for-profit HCFs of various sizes.^11^^,^^15^ After a pilot in one district in 2018, the program scaled up during 2020 and 2021 to include four additional districts during the COVID-19 pandemic; all districts were still part of the program in 2023. In each district, we coordinated with the District Health Office to set up a district ABHR production unit, hired staff to locally produce and distribute ABHRs, and, in most districts, supported the distribution of basic handwashing stations as part of first-line interventions. ABHR was distributed as needed throughout the intervention period,^11^ but after the initial provision, the maintenance, repair, and replacement of ABHR dispensers and handwashing stations were the responsibility of the recipient HCFs.

We assessed whether and to what extent overall access to HH materials and HHA changed from before to after the local ABHR interventions. Growing evidence indicates that the COVID-19 pandemic led to changes in HHA in clinical settings.^16^^,^^17^ The HH observation data collected at multiple time points between 2018 and 2022 provided a unique opportunity to estimate and control for COVID-19 burden as a proxy for risk perception and its changes over time while exploring the impact of the distribution of locally produced ABHRs on HHA. Results from this evaluation can inform decision-making for sustaining, strategizing, and scaling up local production of ABHRs in resource-limited settings.

## MATERIALS AND METHODS

### Study population.

**Figure 1.**
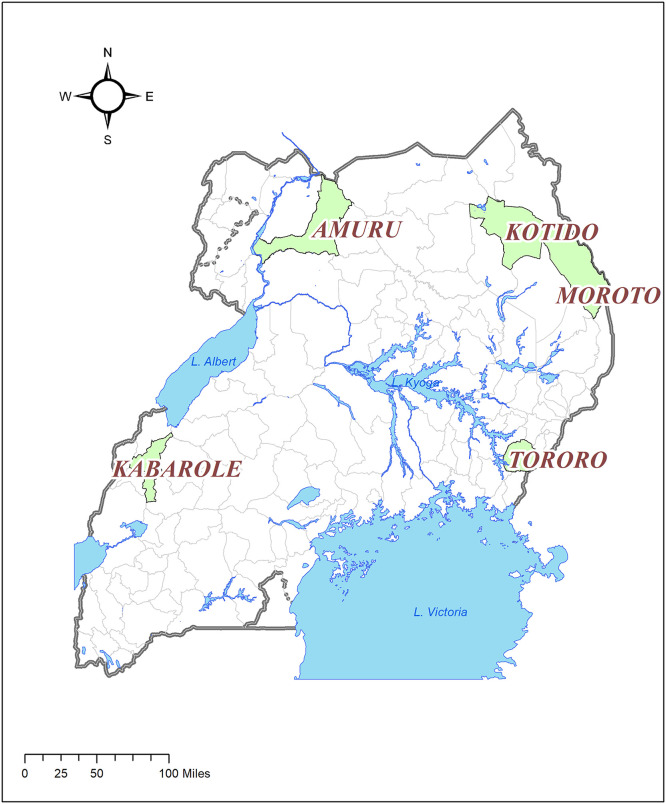
Map of intervention districts in Uganda.

This study was based in five districts in Uganda, where ABHR interventions were initiated at three different time points (see map in Figure 1). In 2018, prior to the onset of the COVID-19 pandemic, Kabarole in western Uganda was selected as a pilot district because of its high public health risk from cross-border population movement with the Democratic Republic of the Congo, which was experiencing and continued to experience periodic outbreaks of Ebola disease.^11^ As part of the emergency response to the COVID-19 pandemic, four additional districts were included.^15^ Amuru, which borders south Sudan to the north, and Tororo, which borders Kenya to the east, were added to the intervention program in late 2020 to early 2021 for their locations along high-volume trucker routes identified as contributors to early clusters of COVID-19.^18^ Moroto and Kotido districts were included in the program in 2021 because of their low-economic status within the country and their challenges in providing WASH resources owing to the semiarid climate, water scarcity, and a nomadic population (Figure 1).

In Kabarole, Moroto, and Kotido, all public and a select number of private HCFs received the intervention. In Amuru and Tororo, the Population Connectivity Across Borders Toolkit^19^ was used to identify HCFs more likely to treat high-risk population groups and thus to be included in the intervention. Healthcare facilities within the Uganda healthcare system are classified into levels based on their catchment areas and the services they provide. The levels of HCFs include Health Center (HC) II, which provides community-based outpatient services; HC III, which additionally includes simple diagnostic and maternal health services; HC IV, which further includes surgical services; and hospitals, which have specialized services in addition to all other services provided by lower-level HCFs.^20^ All levels of HCFs were included in the study.

### Data collection procedures.

**Figure 2.**
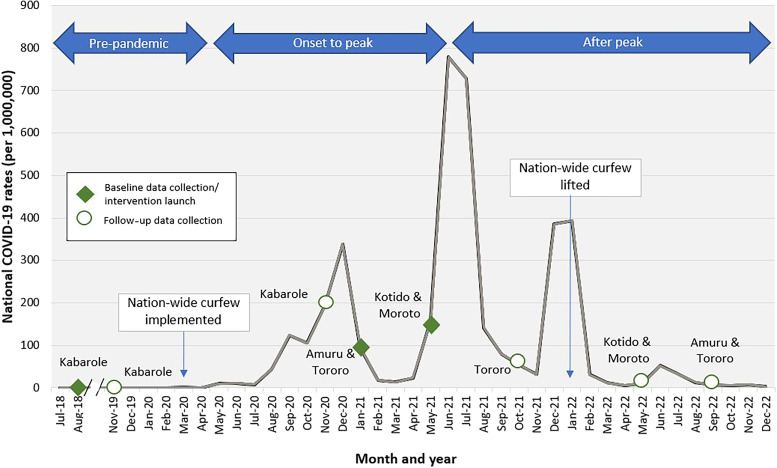
National monthly COVID-19 rates and timing of assessments by district between 2018 and 2022.

Prior to the interventions, we conducted a baseline assessment in each district between August 2018 and May 2021 (Figure 2), including a WASH assessment^21^ for all participating HCFs and observations of HCWs’ use of HH materials in most HCFs. Follow-up evaluations were conducted between November 2019 and September 2022, approximately 12 months after the establishment of the ABHR production unit and distribution system in each district.

### Availability of HH materials.

As part of the HCF WASH assessment, enumerators observed the presence and functionality of HH materials, including handwashing stations and ABHR dispensers, in all rooms where HH materials were required. A functioning handwashing station was defined as having an operational tap, water, and soap. A functioning ABHR station was defined as having an operational dispensing mechanism with ABHR present. Rooms were identified as requiring HH materials if they met at least one of the following criteria: 1) HCWs had physical contact with patients, 2) HCWs handled laboratory specimens or medications, 3) patients stayed overnight, or 4) HCWs put on or removed personal protective equipment (PPE). The coverage of functional HH materials was calculated as the percentage of all rooms requiring HH materials that had either at least one functional handwashing station or ABHR station or both. WASH assessments also included patient and staff volumes and water source and accessibility. Data were collected using mobile data collection platforms including SurveyCTO (Dobility, Inc., Cambridge, MA) or KoboToolbox (Cambridge, MA).

### Hand hygiene adherence among HCWs.

Whether HCWs washed their hands with soap and water, applied ABHR, or did not perform either of these practices during physical interactions with patients was assessed by enumerators’ direct observations of HCWs, using a modified version of the WHO HH observation method^21^ that focused on two of the five moments for HH^22^: before touching a patient and after touching a patient. Enumerators also recorded whether HCWs donned new gloves before patient contact, as past studies indicated that glove use interferes with appropriate HH,^23^^,^^24^ although enumerators did not collect information to assess appropriate glove use. To note, glove use is not considered HHA, as gloves are a type of PPE that serve only as a physical barrier but do not include an aseptic action.^22^

Eligible HCWs were any providers who came into direct physical contact with patients, including doctors, clinical officers, nurses, midwives, and laboratory technicians (recorded by HCW type). In Kabarole, the number of HCWs selected for observation at each HCF was proportional to HCF size: one HCW at HC IIs, three at HC IIIs, and four at HC IVs (no public hospitals were included in Kabarole). When the number of HCWs on duty on the day of the data collection exceeded the target number at a HCF, enumerators randomly selected HCWs from a numbered list of all eligible HCWs present. In other districts, the target number of HCWs observed was set from 8 to 10 per HCF and was selected through convenience sampling, or all HCWs present were selected if there were fewer than the target number.

For each HCW observed, enumerators observed the use of HH materials and gloves for three to five physical contacts with patients or for 20 minutes, whichever came first. Each patient contact comprised two HH opportunities: one before patient contact and the other after patient contact. A new contact began once HCWs saw and had a physical contact with a new patient or had a second contact with the patient after the HCW’s hands touched something other than the patient or medical equipment during the patient encounter. Therefore, multiple physical contacts with the same patient were possible. Enumerators recorded the HH moment (i.e., whether the HH opportunity was before or after patient contact), procedure type (i.e., invasive, defined as the HCW coming in contact with broken skin such as injections, or noninvasive), and contact type (i.e., whether the HH opportunity was at the first contact with the patient or at a subsequent contact).

Data were collected on paper forms and transferred into Microsoft Excel spreadsheets. We restricted the analytic sample to the observations conducted at HCFs where both baseline and at least one follow-up time point were available. We also excluded data collected in June 2019 and December 2022 in Kabarole, when Ebola-related public health threats substantially increased HHA: in June 2019 (when three imported Ebola disease cases were identified in the neighboring district^25^) and in late 2022 (when there was a multi-district Ebola disease outbreak^26^). Applying these criteria, the analytic sample included one follow-up assessment for Amuru, Kotido, and Moroto and two for Tororo and Kabarole.

Enumerators obtained consent from HCF leadership and HCWs to conduct observations. To reduce bias, enumerators did not inform either the HCF leadership or the HCWs that they were specifically observing the use of HH materials and gloves. The protocol and study activities were reviewed and approved by the Centers for Disease Control and Prevention and the Uganda Virus Research Institute (UVRI) and was determined to be program evaluation and not to be human subject research (CDC project ID: 072018MP, GHS000045, UVRI Ref: GC/127/17/02/03).

### Analytical approach.

First, we compared coverage of HH materials in patient care rooms from baseline to follow-up by district. Second, we compared changes in HHA before versus after the interventions by district and other key characteristics. We also examined glove use before patient contact at baseline and follow-up as an auxiliary analysis.

We used logistic regression using generalized estimation equations (GEEs) to explore the association between the intervention and HHA, accounting for clustering among HH opportunities observed for the same HCW. Because the interventions and data collections took place both prior to and at different stages of the COVID-19 pandemic, changes in HHA may have been driven not only by the interventions but also by the changing perceived risk for COVID-19. To control for this potential COVID-19 effect, we categorized the COVID-19 risk burden into three stages: 1) pre–COVID-19 (August 2018–February 2020, used as a reference), 2) COVID-19 onset to the peak in Uganda (March 2020–May 2021), and 3) after the peak (June–September 2022) and included them as a proxy for its changing risk perception in the models (Figure 2). Pre-pandemic HH observation data were limited to the Kabarole district, though they were used to estimate and control for the effect of COVID-19 risk perceptions across all participating districts. The effect of interventions was captured at the facility level as a dichotomous indicator coded 1 for observations conducted during the follow-up and 0 for observations conducted at baseline. We also controlled for HH moment, procedure type, contact type, HCW type, HCF level, and district.

We estimated a base model that explored the overall association between HHA and the interventions (Model 1) as well as models with significant interactive effects of the intervention by district (Model 2) and HCF level (Model 3) to identify where the interventions may or may not have had effects on HHA. All analyses were conducted using STATA v.17 (StataCorp LLC, College Station, TX).

## RESULTS

### Characteristics of HCFs participating in the interventions.

Across the five districts, a total of 92 HCFs participated in the interventions. Of 90 HCFs for which we had 2022 follow-up assessment data, 51% were HC IIIs and 34% were HC IIs (Table 1). Most HCFs used piped water (53%) or boreholes (32%). Water sources were mostly on-site (69%); however, interruptions in water supply were common (45%). After the interventions, most HCFs (89%) stated that they had sufficient ABHR for all needs and did not experience an interruption in supply from the intervention, although half of the facilities in Kotido reported experiencing interruptions. The majority (71%) of HCFs reported additional formal supply lines of ABHR, mostly from the Ministry of Health (58%).

**Table 1:** Characteristics of healthcare facilities participating in interventions by district based on 2022 follow-up assessment

Characteristics	Kabarole (January 2022)		Amuru (August 2022)		Tororo (August 2022)		Kotido (May 2022)		Moroto (May 2022)		Total	
	%/Mean	*n*/Range	%/Mean	*n*/Range	%/Mean	*n*/Range	%/Mean	*n*/Range	%/Mean	*n*/Range	%/Mean	*n*/Range
Facility Type												
HC II	43%	12	0%	0	0	0	36%	8	58%	11	34%	31
HC III	50%	14	88%	7	38%	5	59%	13	37%	7	51%	46
HC IV	7%	2	13%	1	23%	3	5%	1	5%	1	9%	8
Hospital	0%	0	0%	0	38%	5	0%	0	0%	0	6%	5
Total	100%	28	100%	8	100%	13	100%	22	100%	19	100%	90
Administrative Status												
Public	100%	28	63%	5	77%	10	86%	19	79%	15	86%	77
Private, Not for Profit	0%	0	38%	3	0%	0	14%	3	21%	4	11%	10
Private for Profit	0%	0	0%	0	23%	3	0%	0	0%	0	3%	3
Total	100%	28	100%	8	100%	13	100%	22	100%	19	100%	90
Mean Monthly Outpatient Consultations (range)	753	(90–1,463)	1,239	(150–3,360)	2,056	(0–6,000)	237	(5–1,000)	662	(74–4,178)	734	(0–6,000)
Mean Monthly Admissions (range)*	24	(0–135)	136	(12–350)	230	(0–1,400)	13	(0–85)	61	(0–696)	59	(0–1,400)
Mean Clinical Staff (range)	10	(1–78)	14	(5–28)	42	(6–180)	7	(1–33)	21	(3–216)	15	(1–216)
Mean Nonclinical Staff (range)	12	(2–42)	5	(2–7)	13	(1–50)	4	(0–7)	11	(0–144)	8	(0–144)
Main Water Source for Handwashing at the Facility												
Piped Water	71%	20	0%	0	69%	9	41%	9	53%	10	53%	48
Borehole	0%	0	100%	8	31%	4	41%	9	42%	8	32%	29
Rainwater Harvest	21%	6	0%	0	0%	0	18%	4	0%	0	11%	10
Other	7%	2	0%	0	0%	0	0%	0	5%	1	3%	3
Total	100%	28	100%	8	100%	13	100%	22	100%	19	100%	90
Location of the Water Source												
On the Ground of the Facility	86%	24	88%	7	54%	7	45%	10	74%	14	69%	62
Within 500 m of the Facility	7%	2	13%	1	46%	6	32%	7	0%	0	18%	16
More Than 500 m from the Facility	7%	2	0%	0	0%	0	23%	5	26%	5	13%	12
Total	100%	28	100%	8	100%	13	100%	22	100%	19	100%	90
Water Supply												
Ever had Interruptions to Water Supply Used For Handwashing	39%	11	13%	1	46%	6	68%	15	42%	8	45%	41
Mean Months in the Past Year with Disruption (range)	n/a	n/a	3.0	(3–3)	3.0	(1–11)	2.9	(1–7)	7.6	(1–12)	4.7	(1–12)
ABHR Supply												
ABHR Amount Always Sufficient for All Needs	n/a	n/a	100%	8	85%	11	82%	18	95%	18	89%	55
Ever Had interruptions in ABHR Supply from our Program	n/a	n/a	0%	0	0%	0	50%	11	11%	2	21%	13
Formal Supply Line of ABHR Other Than Our Program	n/a	n/a	75%	6	58%	7	73%	16	79%	15	71%	44
ABHR Received from Government	n/a	n/a	63%	5	33%	4	50%	11	84%	16	58%	36

ABHR = alcohol-based hand rub; HC = Health Center; n/a = information was not collected, therefore not available.

* For Kabarole, it is a mean monthly number of outpatients.

### Coverage of HH materials.

Across all participating HCFs, coverage of at least one HH material (either water and soap or ABHR) in the rooms where they were needed increased from 45% at baseline to 73% at follow-up (Table 2). Overall ABHR coverage increased from 32% to 55%. District-level ABHR coverage increased by 20% to 62% points except for Kotido, where coverage decreased from 72% to 44%.

**Table 2:** Coverage of functional HH materials by district: Pre- and postintervention comparison

Type of HH Materials in Rooms	Baseline		Follow-Up		Difference
	%	*n*	%	*n*	%
Total					
Handwashing Stations*	26	118	37	156	11^†^
ABHR Dispensers	32	147	55	233	23^†^
Any HH Materials	45	201	73	271	29^†^
Total	100	449	100	369	–
By District					
Kabarole					
Handwashing Stations	16	27	59	64	43^†^
ABHR Dispensers	5	8	66	71	61^†^
Any HH Materials	19	32	79	85	60^†^
Total	100	170	100	108	–
Amuru					
Handwashing Stations	53	10	38	6	−15
ABHR Dispensers	53	10	94	15	41^‡^
Any HH Materials	68	13	94	15	26
Total	100	19	100	16	–
Tororo					
Handwashing Stations	41	32	31	16	−10
ABHR Dispensers	48	38	79	41	31^†^
Any HH Materials	63	50	81	42	17^§^
Total	100	79	100	52	–
Moroto					
Handwashing Stations	26	35	48	53	22^†^
ABHR Dispensers	43	58	63	70	20^‡^
Any HH Materials	53	71	77	85	24^†^
Total	100	135	100	111	–
Kotido					
Handwashing Stations	30	14	21	17	−10
ABHR Dispensers	72	33	44	36	−28^‡^
Any HH Materials	76	35	54	44	−22^§^
Total	100	46	100	82	–

ABHR = alcohol-based hand rub; HH = hand hygiene. Healthcare facilities for which both baseline and follow-up assessment data were available were included in the analysis. For districts (Kabarole, Amuru, Tororo) where multiple follow-up assessments were implemented, the first follow-up is presented in this table.

* For the purposes of this study, a few handwashing stations with highly chlorinated water but without soap were considered functional, although not recommended for routine hand hygiene.^10^

^†^ *P* <0.001.

^‡^ *P* <0.01.

^§^ *P* <0.05.

### Characteristics of HH opportunities.

The analytic sample had a total of 2,994 observations from 421 HCWs in 58 HCFs across five districts (Table 3). Most observations were conducted around invasive procedures (59%) and for initial patient contacts (83%). Observations were mostly conducted on nurses (37%), laboratory technicians (25%), and midwives (19%), and at HC IIIs (54%).

**Table 3:** Characteristics of hand hygiene opportunities: Pre- and postintervention comparison

Characteristics	Baseline		Follow-Up		Total	
	%	*n*	%	*n*	%	*n*
Hand Hygiene Moment						
Before patient contact	50	678	50	819	50	1,497
After patient contact	50	678	50	819	50	1,497
Total	100	1,356	100	1,638	100	2,994
Type of Procedure						
Invasive	36	490	46	748	41	1,238
Noninvasive	64	866	54	890	59	1,756
Total	100	1,356	100	1,722	100	2,994
Contact Type*						
First Contact	78	800	87	1,416	83	2,216
Subsequent Contact	22	220	13	222	17	442
Total	100	1,020	100	1,638	100	2,658
Healthcare Worker Type						
Doctor	4	60	4	64	4	124
Clinical Officer	15	198	16	242	15	440
Nurse	40	538	34	572	37	1,110
Midwife	17	232	21	344	19	576
Laboratory Technician/Assistant	24	328	25	416	25	744
Total	100	1,356	100	1,638	100	2,994
Healthcare Facility Level						
HC II	16	216	13	226	14	442
HC III	48	656	58	910	54	1,566
HC IV	19	260	18	306	18	566
Hospital	17	224	11	196	14	420
Total	100	1,356	100	1,638	100	2,994
District						
Kabarole	25	334	51	788	37	1,122
Amuru	10	138	5	84	7	222
Tororo	22	302	15	256	19	558
Moroto	31	420	17	286	24	706
Kotido	12	162	13	224	13	386
Total	100	1,356	100	1,722	100	2,994

HC = Health Center.

* Contact type was not collected in Kabarole at baseline. Therefore, *N* = 2,658 for this variable.

### Hand hygiene adherence by key characteristics.

Overall, HHA improved from 22% at baseline to 28% at follow-up (Table 4). Alcohol-based hand rub use increased from 17% to 25%, whereas handwashing with soap decreased from 6% to 2%. The size and direction of change in HHA from baseline to follow-up varied by certain characteristics. Adherence increased from 29% to 35% for HH opportunities after patient contact and from 24% to 29% for those before and after noninvasive procedures. Hand hygiene adherence decreased from 29% to 25% for HH opportunities before and after first contact with a given patient. Hand hygiene adherence among midwives increased from 23% to 39%. Hand hygiene adherence decreased at HC IIs from 25% to 12%, whereas it increased at HC IIIs from 18% to 30%. Kabarole was the only district to have a significant increase in HHA (from 3% to 30%), whereas it decreased in Kotido (40–21%) and Tororo (39–30%).

**Table 4:** Hand hygiene adherence by key characteristics: Pre- and postintervention comparison

Characteristics	Baseline		Follow-Up		Difference
	%	*n*	%	*n*	%
Hand Hygiene Materials Used					
Washed Hands with Soap and Water*	6	78	2	26	−4^†^
Applied ABHR to Hands	17	225	25	410	8^†^
Overall HH Adherence^‡^	22	302	28	435	6^§^
Glove Use (before patient contact only)					
Glove Used Alone	24	160	36	292	12^†^
Glove Used with Appropriate HH	1	13	2	30	1^‖^
Overall Glove Use	26	173	39	322	13^†^
Hand Hygiene Adherence by Key Characteristics					
Hand Hygiene Moment					
Before patient contact	16	108	18	151	3
After patient contact	29	194	35	284	6^‖^
Type of Procedure					
Invasive	19	93	23	173	4
Noninvasive	24	209	29	262	5^‖^
Contact Type					
First Contact	29	233	25	358	−4^‖^
Subsequent Contact	26	58	35	77	8
Healthcare Worker Type					
Doctor	45	27	34	22	−11
Clinical Officer	33	66	37	89	3
Nurse	20	109	23	131	3
Midwife	23	54	39	133	16^†^
Laboratory Technician	14	46	14	60	0
Healthcare Facility Levels					
HC II	25	53	12	27	−13^§^
HC III	18	120	30	272	12^†^
HC IV	28	73	28	85	0
Hospital	25	56	26	51	1
District					
Kabarole	3	11	30	240	27^†^
Amuru	40	55	44	37	4
Tororo	39	118	30	78	−9^‖^
Moroto	13	54	12	33	−1
Kotido	40	64	21	47	−19^†^

ABHR = alcohol-based hand rub; HC = Health Center; HH = hand hygiene. All the follow-up data from multiple assessments were combined. *P*-values refer to the difference between baseline and follow-up based on χ^2^ test.

* This includes three hand hygiene opportunities where healthcare workers used highly chlorinated water.

^†^ *P* <0.001.

^‡^ The sum of observations where hands were washed and ABHR was applied do not sum up to the total because healthcare workers washed hands with soap and used ABHR in two hand hygiene opportunities.

^§^ *P* <0.01.

^‖^ *P* <0.05.

Donning gloves before patient contact, which is not part of HHA calculations, was 26% at baseline and 39% at follow-up. Only 1–2% of glove use was accompanied by prior use of ABHR or handwashing with soap and water. The largest increases in glove use were among nurses and laboratory technicians, at HC IIs, and in Kotido (Supplemental Table 1).

### Logistic regression models using GEEs and exploring changes in HHA.

In Model 1, we assessed the average change in HHA after versus before the interventions, adjusted for COVID-19 burden and other covariates from Table 4 and without interactions. The odds of HHA at follow-up were 4.6 times greater after the intervention than at baseline (95% CI: 1.8–11.8; Table 5). The odds of HHA during the period between the onset of the pandemic and the disease peak was 4.0 (95% CI: 2.2–7.2) times greater than pre-pandemic, whereas HHA post-peak was comparable to pre-pandemic (adjusted odds ratio = 0.5; 95% CI: 0.1–2.0). Model 2 included interactions to estimate district-specific changes in HHA after versus before interventions and showed that all districts, excluding Kotido, had higher odds of HHA at follow-up than at baseline. Model 3 included interactions to estimate HCF level–specific changes in HHA after versus before interventions and showed that HCWs in HC III, HC IV, and hospitals, but not in HC IIs, had higher odds of HHA after interventions than at baseline.

**Table 5:** Hand hygiene adherence: Adjusted odds ratios from logistic regression using generalized estimation equations

Independent Variables	Model 1 (Base Model)		Model 2 (Interactive Model)		Model 3 (Interactive Model)	
	Adj. Odds Ratios	95% CI	Adj. Odds Ratios	95% CI	Adj. Odds Ratios	95% CI
Interventions	4.56*	(1.77–11.79)	–	–	–	–
Hand Hygiene Moment and Type of Procedure						
Before Any Procedure	Ref.	Ref.	Ref.	Ref.	Ref.	Ref.
After Invasive Procedure	3.55^†^	(2.83–4.47)	3.54^†^	(2.81–4.45)	3.56^†^	(2.83–4.48)
After Noninvasive Procedure	2.26^†^	(1.83–2.79)	2.08^†^	(1.72–2.51)	2.07^†^	(1.72–2.50)
Contact Type^‡^						
First Contact	1.21	(0.95–1.53)	1.200	(0.94–1.53)	1.210	(0.95–1.53)
Subsequent Contact	Ref.	Ref.	Ref.	Ref.	Ref.	Ref.
Healthcare Worker Type						
Doctor	4.01^†^	(1.92–8.37)	4.37^†^	(2.07–9.20)	3.96^†^	(1.90–8.26)
Clinical Officer	5.24^†^	(3.11–8.83)	5.00^†^	(2.96–8.45)	5.07^†^	(3.01–8.53)
Nurse	2.24^†^	(1.44–3.48)	2.15*	(1.38–3.35)	2.17*	(1.40–3.37)
Midwife	3.18^†^	(1.96–5.17)	3.12^†^	(1.92–5.11)	3.16^†^	(1.94–5.13)
Laboratory Technician/Assistant	Ref.	Ref.	Ref.	Ref.	Ref.	Ref.
Healthcare Facility Level						
HC II	Ref.	Ref.	Ref.	Ref.	Ref.	Ref.
HC III	1.07	(0.63–1.81)	1.05	(0.62–1.80)	0.65	(0.33–1.28)
HC IV	1.03	(0.56–1.90)	1.04	(0.56–1.92)	0.75	(0.33–1.69)
Hospital	0.93	(0.47–1.83)	0.91	(0.46–1.80)	0.52	(0.22–1.21)
Healthcare Facility Levels × Interventions						
HCII	–	–	–	–	1.57	(0.41–6.02)
HCIII	–	–	–	–	4.90*	(1.87–12.88)
HCIV	–	–	–	–	3.62^§^	(1.16–11.37)
Hospital	–	–	–	–	5.78*	(1.59–20.99)
District						
Kabarole	1.23	(0.40–3.78)	1.33	(0.42–4.21)	1.33	(0.43–4.11)
Amuru	5.12^†^	(2.73–9.60)	4.03^†^	(1.81–8.97)	5.27	(2.81–9.87
Tororo	4.23^†^	(2.49–7.19)	4.38^†^	(2.21–8.67)	4.23	(2.49–7.19)
Moroto	Ref.	Ref.	Ref.	Ref.	Ref.	Ref.
Kotido	3.73^†^	(2.23–6.26)	5.79^†^	(2.88–11.81)	3.96	(2.24–7.11)
District × Interventions						
Kabarole	–	–	4.58*	(1.77–11.81)	9.06*	(2.56–32.03)
Amuru	–	–	5.52*	(1.13–5.95)	17.32*	(3.17–94.46)
Tororo	–	–	2.59^§^	(1.15–7.50)	5.10^§^	(1.41–18.38)
Moroto	–	–	2.94^§^	(1.15–7.50)	6.86*	(1.81–25.96)
Kotido	–	–	1.19	(0.47–3.00)	1.69	(0.41–6.99)
COVID-19 Pandemic Stage						
Stage 0 (pre-pandemic)	Ref.	Ref.	Ref.	Ref.	Ref.	Ref.
Stage 1 (onset to peak)	4.02^†^	(2.24–7.19)	4.01^†^	(3.82–23.35)	9.48^†^	(3.83–23.46)
Stage 2 (peak to present)^¶^	0.54	(0.14–2.01)	–	–	0.640	(0.16–2.46)
Residual Intraclass Correlation	0.27	–	0.27	–	0.27	–

Adj. = adjusted; HC = Health Center; OR = odds ratio; Ref. = reference.

* *P* <0.01.

^†^ *P* <0.001.

^‡^ The OR and CI for contact type were obtained from a model applied on the sample excluding Kabarole baseline, which did not have this information.

^§^ *P* <0.05.

^¶^ Stage 2 of the COVID-19 pandemic was dropped from Model 3 owing to collinearity with interaction between interventions and districts.

## DISCUSSION

Our study observed an overall increase in HH materials at points of care after the implementation of local ABHR production and distribution interventions.^11^^,^^15^ Postintervention, access to ABHRs, rather than handwashing stations, was the driver of improved coverage of HH materials. We also observed a small, albeit statistically significant, overall increase in HHA, which was also driven by increased use of ABHRs. Our multivariate analysis controlled for the pandemic effects and estimated that the average odds of adhering to appropriate HH was about 4.6 times greater after the interventions across districts. The increase in ABHR availability and the subsequent increase in HHA suggest that district-led efforts to produce ABHRs locally at-scale for district-wide distribution and monitoring schemes to maintain supplies of ABHR for use within HCFs were successful. These results are similar, albeit on a district-wide scale versus a facility-level scale, to those observed by Ndegwa et al.,^14^ which showed a smaller increase in HHA after hospital-based production of ABHRs.

However, disaggregation of the data by districts revealed a complex local context and secular differences in coverage of HH materials and HHA based on when data collection occurred. The observed increases in both ABHR coverage and HHA were most evident in Kabarole, likely because the baseline was conducted prior to the pandemic in 2018, when ABHR was not very common in Uganda, whereas the second follow-up was in 2020 during an initial phase of the pandemic with heightened HH awareness and risk perception. Other districts had higher-than-expected baseline levels of observed ABHR coverage and HHA likely because the baselines were conducted during the early stage of the pandemic and HCFs were likely already receiving HH materials from other sources. In most districts, however, our intervention appeared to contribute to an increase in, or at least sustained the level of, observed ABHR coverage and HHA, even after the pandemic passed its peak.

The exception was Kotido, where we documented significant decreases in both observed coverage of HH materials, particularly of ABHR, and HHA after the intervention. This is likely because HCFs in Kotido received ABHRs separately from our intervention as part of an intensive, 6-month infection prevention control training program that ended just before the baseline assessment for our program in May 2021, resulting in the substantially elevated coverage of HH materials and HHA at baseline. This was coupled with the follow-up assessment being conducted in the midst of a resurgence of armed conflicts in 2022 in the district,^27^ which resulted in political insecurity, road closures, and the disruption of ABHR distribution from our intervention and other sources.

Increases in HHA after interventions were more evident in larger HCFs, such as HC IIIs, HC IVs, and hospitals. This may be related to the finding that HCWs were more likely to adhere to HH after invasive procedures: If the perceived need for appropriate HH among HCWs is elevated where specialized procedures, including surgeries and emergency obstetric care, take place, then HHA may be greater in higher-level facilities that provide these services compared with HC IIs, which provide community-based preventive and promotive health services. Thus, facilitating more access through the free distribution of ABHRs may have induced more immediate improvement in HHA among the former than the latter. Consistent with past studies,^28^ HCWs were significantly more likely to adhere to HH after patient contact, particularly after invasive procedures, and this disparity did not change after the interventions. As shown elsewhere,^29^^,^^30^ this seems to suggest that HHA among HCWs may be consistently motivated more by their desire to protect themselves rather than to protect patients from potential infection. Healthcare worker education on the five key moments for HH^31^ should continue to ensure best practices in the use of HH resources.

Limited HHA at HC IIs at follow-up and among laboratory technicians and nurses, which did not appear to change markedly from baseline to follow-up, is likely due in part to increased use of gloves. Past studies have shown that overreliance on gloves is associated with poor HHA.^23^^,^^24^ We similarly found that gloves were rarely used in combination with prior appropriate HH at both baseline and follow-up. Use of gloves does not replace handwashing with soap or application of ABHR in healthcare settings, and donning gloves without performing HH beforehand is not considered appropriate HH.^32^ Education on HH and the appropriate use of gloves should be encouraged, especially among nurses and laboratory technicians.

Finally, we observed a decrease in handwashing with soap after the interventions despite the distribution of handwashing stations and the subsequent increase in their availability. Healthcare workers appear to prefer using ABHRs to handwashing with soap, likely owing to the convenience and short application time of ABHRs,^13^^,^^33^ especially in rural areas with a high patient-to-HCW ratio and frequent water shortages, and ABHR is the gold standard for routine HH in the HCF context.^10^ However, it is important to continue efforts to improve access to clean water and ensure access to handwashing stations because handwashing with soap is recommended when hands are visibly dirty, soiled with blood or body fluids, and after using the toilet.

This study had several limitations. First, pre-pandemic assessment data used to control for pandemic-related changes in HHA were available in only one district, Kabarole, necessitating assumptions that the levels of pre-pandemic HHA and the size of the effects of the pandemic on HHA in other districts were similar to those in Kabarole. Relatedly, the categorical measure of the COVID-19 pandemic may not have captured the full extent of its impact on HHA. Secondly, given the challenges associated with data collection during the pandemic response, we were unable to collect both baseline and follow-up data from all 92 participating HCFs, and convenience sampling of HCWs for HHA observations was required within some higher-level HCFs, limiting the representativeness of results. In addition, a large proportion of participating HCFs reported receiving ABHRs from other sources in addition to our intervention; thus, increases in HHA may not have been due to our interventions alone. Finally, although the enumerators for this study received in-person or virtual training on the use of the tools prior to each data collection, they were not able to be validated as recommended by the WHO^10^ because of the data collection being conducted within pandemic response activities.

## CONCLUSION

This study explored changes in the availability of HH materials and HHA after ABHR interventions, in particular, adjusting for the effects of the COVID-19 pandemic on HHA. We documented increases in access to and use of HH materials; however, HHA remained low overall at 28% at follow-up. Although local production and distribution of ABHRs at the district scale remains a promising way to increase the availability of HH materials, additional strategies, including training and education of HCWs and creation of a HH safety culture as outlined in the WHO multimodal HH improvement strategy, could further improve HHA.^8^

## Supplemental Materials

10.4269/ajtmh.24-0040 Supplemental Materials
