# Supplementary material for: Changes in Access to Alcohol-Based Hand Rub and Hand Hygiene Adherence among Healthcare Workers after a Hand Rub Production and Distribution Program in Rural Uganda before and during the COVID-19 Pandemic
Source: Am J Trop Med Hyg. 2024 Sep 18;111(6):1343–52. doi: 10.4269/ajtmh.24-0040 (PMC11619488; doi:10.4269/ajtmh.24-0040)
Supplement: Supplemental Materials [file tpmd240040.SD1.pdf]

Supplementary Table 1

Use of gloves before patient contact: Pre- and post-intervention comparison

	Baseline		Follow-Up		Difference
**By Key Characteristics**	%	*n*	%	*n*	%
**Type of procedure**					
Invasive	65%	157	69%	259	5%
Non-invasive	4%	16	12%	54	8%***
**Contact type**					
First contact	25%	101	40%	281	14%***
Subsequent contact	15%	16	29%	32	14%*
**Healthcare worker type**					
Doctor	17%	5	31%	10	15%
Clinical officer	9%	9	7%	9	-2%
Nurse	10%	28	32%	92	22%***
Midwife	19%	22	12%	21	-7%
Lab technician	67%	109	87%	181	20%***
**Healthcare facility levels**					
HC II	5%	5	43%	49	29%***
HC III	27%	87	37%	167	10%**
HC IV	28%	37	41%	63	13%*
Hospital	39%	44	35%	34	-5%
**District**					
Kabarole	34%	56	37%	144	2%
Amuru	25%	17	48%	20	23%*
Tororo	36%	55	33%	42	-4%
Moroto	15%	31	26%	37	11%**
Kotido	17%	14	63%	70	45%***

Notes:

The analytic sample comprises 1,494 hand hygiene opportunities before patient contact in the follow-up data from four districts exluding Kabarole, where information on glove use was insufficient.

P* <0.05; ** *P* <0.01; * *P* <0.001 refer to the difference between baseline and follow-up based on the chi-square test.

An auxiliary analysis of a limited sample of 425 hand hygiene opportunities before patient contact with information on glove reuse showed that reusing gloves was frequent: 18% (75 opportunities) were recorded as using new gloves while 24% (103 opportunities) as reusing the same gloves.
